# Crystal structure of (5*R*)-5-[(1*S*)-1,2-di­hydroxy­eth­yl]-4-meth­oxy-3-phenyl-2,5-di­hydro­furan-2-one

**DOI:** 10.1107/S1600536814021370

**Published:** 2014-09-30

**Authors:** Santosh R. Kote, Shankar R. Thopate, Sushil K. Gupta, Ray J. Butcher

**Affiliations:** a Department of Chemistry, Post Graduate School for Biological Studies, Ahmednagar College, Ahmednagar 414 001, India; b School of Studies in Chemistry, Jiwaji University, Gwalior 474 011, India; c Department of Chemistry, Howard University, 525 College Street NW, Washington, DC 20059, USA

**Keywords:** Crystal structure, l-ascorbic acid derivative, hydrogen bonding., crystal structure

## Abstract

In the title compound, C_13_H_14_O_5_, the furan ring is essentially planar [maximum deviation = 0.031 (3) Å] with a stereogenic center (*R*) at the *sp* ^3^ hybridized C atom. The C atom bearing the dihy­droxy ethyl group is *S*. The absolute configuration is based on the precursor in the synthesis. The two O—H groups are in an *anti* conformation with respect to each other. The mean plane of the furan­one group is twisted by 8.2 (4)° from that of the phenyl ring. In the crystal, mol­ecules are linked by O—H⋯O hydrogen bonds involving furan­one C=O groups and symmetry-related hy­droxy groups, forming a two-dimensional network parallel to (001). Weak C—H⋯O hydrogen bonds are observed within the two-dimensional network.

## Related literature   

For the biological activity of 5,6-*O*-modified and 2,3-di-*O*-alkyl derivatives of l-ascorbic acid, see: Tanuma *et al.* (1993 ▶); Gazivoda *et al.* (2007 ▶); Wittine *et al.* (2012 ▶); Kote *et al.* (2014 ▶). For related structures, see: Koo & McDonald (2005 ▶); Tanaka *et al.* (1986 ▶); Sugimura (1990 ▶). For a description of the Cambridge Structural Database, see: Allen (2002 ▶).
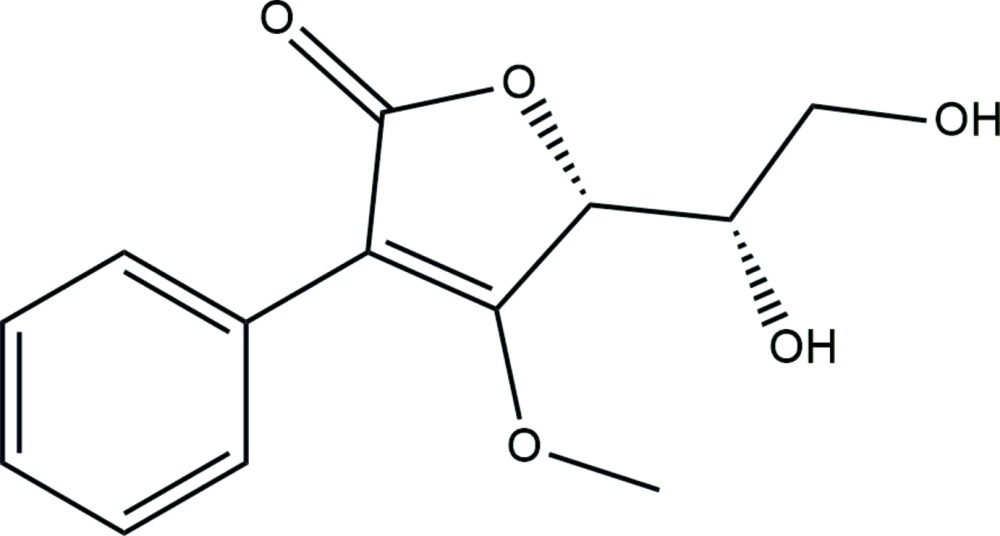


## Experimental   

### Crystal data  

C_13_H_14_O_5_
*M* *_r_* = 250.24
Monoclinic, 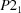

*a* = 7.5110 (5) Å
*b* = 4.9298 (3) Å
*c* = 16.6625 (16) Å
β = 93.268 (6)°
*V* = 615.97 (8) Å^3^
*Z* = 2
Mo *K*α radiation
μ = 0.10 mm^−1^
*T* = 293 K
0.4 × 0.3 × 0.08 mm

### Data collection  

Agilent Xcalibur, Ruby, Gemini diffractometer
Absorption correction: multi-scan (*CrysAlis PRO*; Agilent, 2011 ▶) *T*_min_ = 0.714, *T*_max_ = 1.000
8346 measured reflections
1243 independent reflections
655 reflections with *I* > 2σ(*I*)
*R*_int_ = 0.034

### Refinement  

*R*[*F* ^2^ > 2σ(*F* ^2^)] = 0.059
*wR*(*F* ^2^) = 0.126
*S* = 1.05
1243 reflections
166 parameters
1 restraint
H-atom parameters constrained
Δρ_max_ = 0.13 e Å^−3^
Δρ_min_ = −0.15 e Å^−3^

### 

Data collection: *CrysAlis PRO* (Agilent, 2011 ▶); cell refinement: *CrysAlis PRO*; data reduction: *CrysAlis PRO*; program(s) used to solve structure: *SHELXS97* (Sheldrick, 2008 ▶); program(s) used to refine structure: *SHELXL2014*/6 (Sheldrick, 2008 ▶); molecular graphics: *SHELXTL* (Sheldrick, 2008 ▶); software used to prepare material for publication: *SHELXTL* (Sheldrick, 2008 ▶).

## Supplementary Material

Crystal structure: contains datablock(s) global, I. DOI: 10.1107/S1600536814021370/lh5729sup1.cif

Structure factors: contains datablock(s) I. DOI: 10.1107/S1600536814021370/lh5729Isup2.hkl

Supporting information file. DOI: 10.1107/S1600536814021370/lh5729Isup3.cml

**Fig. 1.**
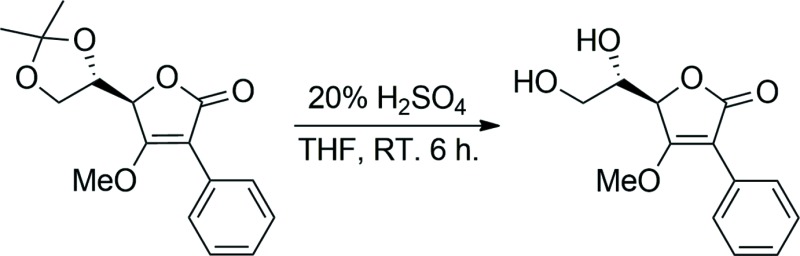
Scheme showing the synthesis of the title compound.

. DOI: 10.1107/S1600536814021370/lh5729fig1.tif
Scheme showing the synthesis of the title compound.

**Fig. 2.**
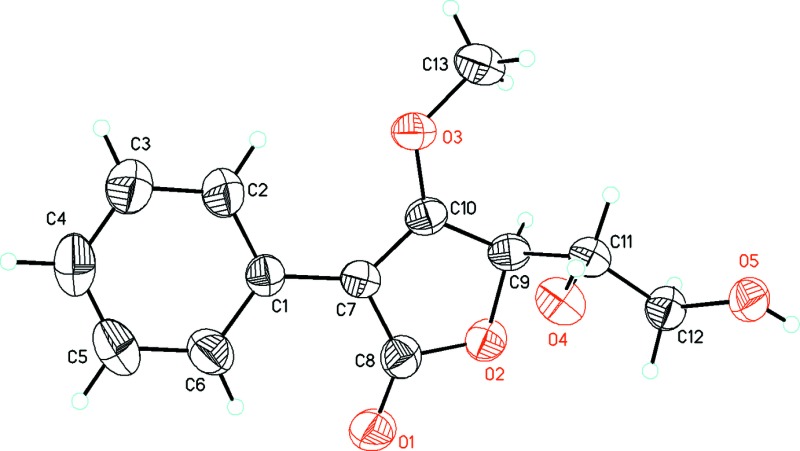
The molecular structure of (I) showing 50% probability displacement ellipsoids.

**Fig. 3.**
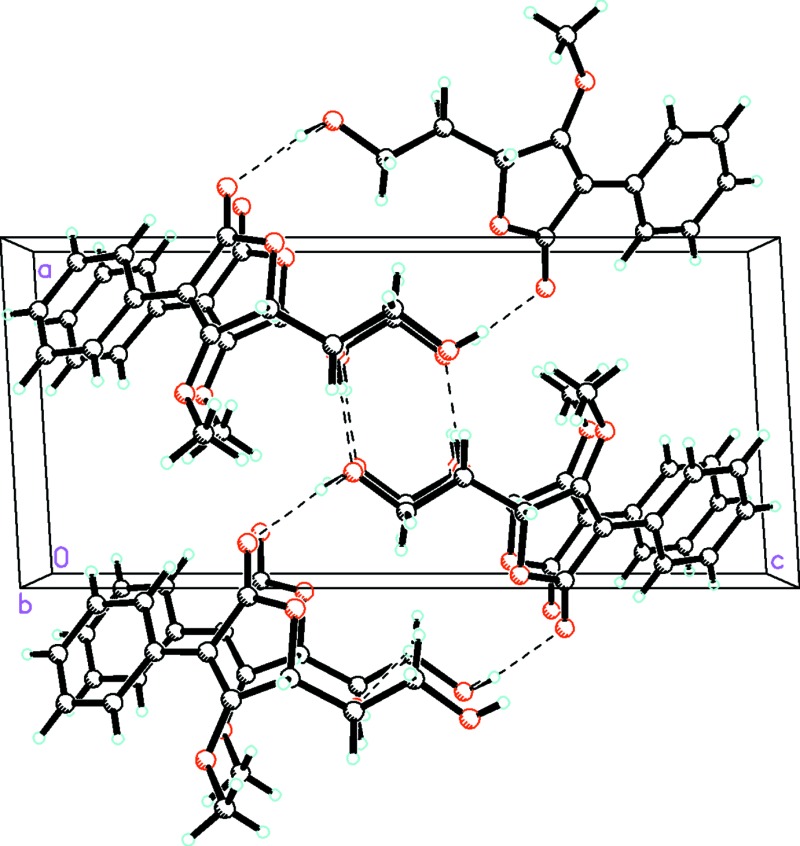
The molecular packing of the title compound, viewed along the b-axis, showing two-dimensional network parallel to (001). Dashed lines indicate hydrogen bonds.

. DOI: 10.1107/S1600536814021370/lh5729fig2.tif
The mol­ecular structure of (I) showing 50% probability displacement ellipsoids.

b . DOI: 10.1107/S1600536814021370/lh5729fig3.tif
The mol­ecular packing of the title compound, viewed along the *b*-axis, showing two-dimensional network parallel to (001). Dashed lines indicate hydrogen bonds.

CCDC references: 998610, 1026319

Additional supporting information:  crystallographic information; 3D view; checkCIF report

## Figures and Tables

**Table 1:** Hydrogen-bond geometry (Å, °)

*D*—H⋯*A*	*D*—H	H⋯*A*	*D*⋯*A*	*D*—H⋯*A*
O4—H4⋯O5^i^	0.82	1.89	2.707 (5)	178
O5—H5⋯O1^ii^	0.82	1.94	2.741 (6)	165
C12—H12*B*⋯O4^iii^	0.97	2.58	3.365 (8)	139

Symmetry codes: (i) 
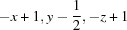
; (ii) 
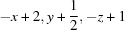
; (iii) 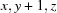.

## supplementary crystallographic information

### S1. Comment

5,6-O-modified L-ascorbic acid derivatives have been found to be effective anti-tumor agents for various human cancers, and induce apoptosis in tumor cells (Tanuma *et al.*, 1993; Gazivoda *et al.*, 2007; Wittine *et al.*, 2012). We have recently reported that 2,3-di-O-alkyl derivatives of 5,6-O-iso­propyl­idene-L-ascorbic acid exhibit anti­cancer activity against human breast cancer cell line (MCF-7), leukemic cell line (HL-60) and cervical cell line (HeLa) (Kote *et al.*, 2014). A search of the Cambridge Structural Database (Version 5.35, updates to November 2013, Allen, 2002) revealed the crystal structures of four related compounds, *viz.* di­methyl-2',3,3', 3a',4,4a,5',6',6a',9a-deca­hydro-6'-hy­droxy-1,3a',7,8,9a-penta­meth­oxy-2',10- dioxo-1,4-ethano-1H-pyrano(3,4-b)benzo­furan-3-spiro-3'-furo(3,2-b)furan-4,5- di­carboxyl­ate (Tanaka *et al.*, 1986); 2,2-di­methyl-7-meth­oxy-1,3,6- trioxa-8-phenyl-4-(2,2-di­methyl-1,3-dioxa­cyclo­propan-4-yl)bi­cyclo­(4.3.0)nonane (Sugimura, 1990); 3,6-di­hydroxy-7-meth­oxy-5-methyl-3-phenyl­hexa­hydro-2H-furo (3,2-b)pyran-2-one methanol solvate and 3,6,7-tri­hydroxy-5-methyl-3-phenyl- hexa­hydro-2H-furo(3,2-b)pyran-2-one (Koo & McDonald, 2005). In view of the importance of the title compound, (I), herein we report its synthesis and crystal structure.

In the title compound (Fig. 2) the furan­one ring is essentially planar [maximum atomic deviation = 0.031 (3) Å] with a stereogenic center (*R*) at atom C9 and (*S*) at atom C11, which bears the di­hydroxy ethyl group. The two O—H groups are in an *anti* conformation with respect to each other, as reflected by torsion angles O5—C12—C11—C9 of 170.5 (6)° and O4—C11—C12—O5 of -69.4 (6)°. The C—C, C_aromatic_—C_aromatic_, C—O and C═O bond lengths in (I) are within their normal ranges. The mean plane of the furan ring (C7/C8/O2/C9/C10) is twisted by 8.2 (4)° from that of the phenyl ring (C1–C6). In the crystal, molecules are linked by inter­molecular O—H···O hydrogen bonds involving furan­one C═O groups and symmetry-related hy­droxy groups (Fig. 3, Table 1) to form a two-dimensional network paralllel to (001). Weak C—H···O hydrogen bonds are observed within the two-dimensional network.

### S2. Experimental

Referring to Fig. 1, to a solution of (*R*)-5-((*S*)-2,2-di­methyl-1,3-dioxolan- 4-yl)-4-meth­oxy-3-phenyl­furan-2-(5H)-one (0.570 g) in 5.0 mL THF was added 2.00 mL of 20% H_2_SO_4_ at room temperature. The reaction mixture was stirred for 6 h at room temperature before it was quenched with NaHCO_3_ solution. The organic layer was extracted with ethyl acetate (3 × 10 mL), combined organic layer was dried over anhydrous Na_2_SO_4_, concentrated under vacuum and eluted through a silica column using a mixture of hexane and ethyl acetate (2:3) as an eluent to afford a white solid. Yield: 0.447 g (91%); HRMS: *m/z* = 251.0919 (calculated), *m/z* = 251.0927 [MH^+^] (found). ^1^H NMR (DMSO-d_6_ + CDCl_3_): δ [ppm] = 6.90 (d, *J* = 7.2 Hz, 2H, Ar—H), 6.71 (m, 3H, Ar—H), 4.44 (s, 1H, C4—H), 4.14 (d, *J* = 6.0 Hz, 1H, C6—H), 3.95 (t, *J* = 6 Hz, 1H, C6—H), 3.43 (m, 1H, C5—H), 3.21 (s, 3H, -OCH3). ^13^C NMR (DMSO-d_6_ + CDCl_3_): δ [ppm] = 168.5, 168.4, 125.4, 125.0, 123.3, 123.0, 100.1, 72.4, 65.2, 58.2, 55.4. IR (KBr): 3349, 3268, 2958, 2920, 1713, 1628, 1465, 1312, 980, 781 cm^-1^. [α]_D_^25^ +2.43° (c 0.28, MeOH). X-ray quality crystals were grown by slow evaporation of a solution of the title compound in a mixture of ethyl acetate and hexane.

### S3. Refinement

H atoms were placed in geometrically idealized positions and constrained to ride on their parent atoms with C—H distances of 0.93–0.98 Å, O—H = 0.82Å and with *U*_iso_(H) = 1.2–1.5 *U*_eq_(C). In the absence of anomalous dispersion effects the Friedel pairs were merged before refinement. The absolute configuration is based on the precursor in the synthesis.

### Crystal data

**Table:**

C_13_H_14_O_5_	*F*(000) = 264
*M**_r_* = 250.24	*D*_x_ = 1.349 Mg m^−^^3^
Monoclinic, *P*2_1_	Mo *K*α radiation, λ = 0.71073 Å
*a* = 7.5110 (5) Å	Cell parameters from 613 reflections
*b* = 4.9298 (3) Å	θ = 3.6–28.4°
*c* = 16.6625 (16) Å	µ = 0.10 mm^−^^1^
β = 93.268 (6)°	*T* = 293 K
*V* = 615.97 (8) Å^3^	Plate, colorless
*Z* = 2	0.4 × 0.3 × 0.08 mm

### Data collection

**Table:**

Agilent Xcalibur, Ruby, Gemini diffractometer	1243 independent reflections
Radiation source: Enhance (Mo) X-ray Source	655 reflections with *I* > 2σ(*I*)
Graphite monochromator	*R*_int_ = 0.034
Detector resolution: 10.5081 pixels mm^-1^	θ_max_ = 25.3°, θ_min_ = 3.6°
ω scans	*h* = −9→9
Absorption correction: multi-scan (*CrysAlis PRO*; Agilent, 2011)	*k* = 0→5
*T*_min_ = 0.714, *T*_max_ = 1.000	*l* = 0→19
8346 measured reflections	

### Refinement

**Table:**

Refinement on *F*^2^	Primary atom site location: structure-invariant direct methods
Least-squares matrix: full	Secondary atom site location: difference Fourier map
*R*[*F*^2^ > 2σ(*F*^2^)] = 0.059	Hydrogen site location: inferred from neighbouring sites
*wR*(*F*^2^) = 0.126	H-atom parameters constrained
*S* = 1.05	*w* = 1/[σ^2^(*F*_o_^2^) + (0.039*P*)^2^] where *P* = (*F*_o_^2^ + 2*F*_c_^2^)/3
1243 reflections	(Δ/σ)_max_ < 0.001
166 parameters	Δρ_max_ = 0.13 e Å^−^^3^
1 restraint	Δρ_min_ = −0.15 e Å^−^^3^

### Special details

**Table:**

Geometry. All e.s.d.'s (except the e.s.d. in the dihedral angle between two l.s. planes) are estimated using the full covariance matrix. The cell e.s.d.'s are taken into account individually in the estimation of e.s.d.'s in distances, angles and torsion angles; correlations between e.s.d.'s in cell parameters are only used when they are defined by crystal symmetry. An approximate (isotropic) treatment of cell e.s.d.'s is used for estimating e.s.d.'s involving l.s. planes.

### Fractional atomic coordinates and isotropic or equivalent isotropic displacement parameters (Å^2^)

**Table:**

	*x*	*y*	*z*	*U*_iso_*/*U*_eq_	
O1	1.1330 (5)	0.1548 (13)	0.2979 (3)	0.0999 (17)	
O2	0.9642 (4)	0.4569 (9)	0.3574 (3)	0.0705 (13)	
O3	0.5525 (5)	0.4727 (10)	0.2375 (3)	0.0830 (14)	
O4	0.6722 (4)	0.1838 (8)	0.4222 (3)	0.0727 (13)	
H4	0.5683	0.1386	0.4272	0.109*	
O5	0.6695 (4)	0.5242 (11)	0.5641 (2)	0.0776 (14)	
H5	0.7173	0.5875	0.6054	0.116*	
C1	0.8238 (8)	0.0846 (12)	0.1705 (4)	0.0609 (16)	
C2	0.6797 (10)	0.0982 (15)	0.1143 (5)	0.090 (2)	
H2A	0.5888	0.2215	0.1225	0.108*	
C3	0.6670 (12)	−0.0633 (19)	0.0476 (5)	0.107 (3)	
H3A	0.5686	−0.0482	0.0114	0.128*	
C4	0.7970 (13)	−0.2454 (16)	0.0338 (5)	0.098 (3)	
H4A	0.7883	−0.3561	−0.0114	0.117*	
C5	0.9404 (11)	−0.2637 (16)	0.0873 (6)	0.100 (3)	
H5A	1.0301	−0.3882	0.0783	0.120*	
C6	0.9552 (9)	−0.1003 (15)	0.1549 (5)	0.085 (2)	
H6A	1.0551	−0.1154	0.1903	0.102*	
C7	0.8339 (7)	0.2571 (12)	0.2420 (4)	0.0565 (17)	
C8	0.9895 (8)	0.2716 (15)	0.2967 (4)	0.071 (2)	
C9	0.7819 (6)	0.5560 (13)	0.3479 (4)	0.0609 (16)	
H9	0.7814	0.7541	0.3428	0.073*	
C10	0.7144 (7)	0.4319 (12)	0.2723 (4)	0.0602 (17)	
C11	0.6847 (7)	0.4704 (14)	0.4227 (4)	0.0584 (16)	
H11A	0.5640	0.5469	0.4189	0.070*	
C12	0.7805 (7)	0.5703 (14)	0.4989 (4)	0.0681 (18)	
H12A	0.8925	0.4742	0.5081	0.082*	
H12B	0.8061	0.7624	0.4944	0.082*	
C13	0.4443 (8)	0.6955 (16)	0.2630 (4)	0.094 (2)	
H13A	0.3670	0.7554	0.2186	0.142*	
H13B	0.5201	0.8424	0.2812	0.142*	
H13C	0.3739	0.6366	0.3060	0.142*	

### Atomic displacement parameters (Å^2^)

**Table:**

	*U*^11^	*U*^22^	*U*^33^	*U*^12^	*U*^13^	*U*^23^
O1	0.061 (2)	0.158 (5)	0.081 (4)	0.027 (3)	0.005 (2)	0.004 (3)
O2	0.054 (2)	0.090 (3)	0.068 (3)	−0.011 (2)	0.003 (2)	0.000 (3)
O3	0.071 (2)	0.092 (3)	0.083 (4)	0.024 (3)	−0.018 (2)	−0.017 (3)
O4	0.066 (2)	0.060 (3)	0.094 (4)	−0.006 (2)	0.016 (2)	0.002 (2)
O5	0.060 (2)	0.113 (4)	0.061 (3)	0.005 (2)	0.011 (2)	0.006 (3)
C1	0.069 (4)	0.056 (4)	0.058 (5)	0.000 (3)	0.007 (4)	0.001 (4)
C2	0.109 (5)	0.084 (6)	0.077 (6)	0.017 (5)	0.000 (5)	−0.015 (5)
C3	0.125 (6)	0.104 (7)	0.090 (7)	0.010 (6)	−0.008 (5)	−0.019 (6)
C4	0.148 (7)	0.077 (6)	0.070 (7)	−0.009 (6)	0.025 (6)	−0.011 (5)
C5	0.110 (6)	0.086 (6)	0.107 (8)	0.013 (5)	0.039 (6)	−0.014 (6)
C6	0.079 (4)	0.087 (6)	0.091 (6)	0.014 (4)	0.015 (4)	−0.005 (5)
C7	0.053 (3)	0.056 (4)	0.061 (5)	0.001 (3)	0.007 (3)	0.007 (4)
C8	0.063 (4)	0.087 (6)	0.064 (5)	−0.003 (4)	0.012 (4)	0.004 (4)
C9	0.056 (3)	0.058 (4)	0.068 (5)	−0.002 (3)	−0.001 (3)	0.006 (4)
C10	0.054 (3)	0.062 (4)	0.063 (5)	−0.004 (4)	−0.002 (3)	0.006 (4)
C11	0.051 (3)	0.056 (4)	0.068 (5)	−0.005 (3)	0.007 (3)	0.002 (4)
C12	0.061 (3)	0.077 (4)	0.066 (5)	−0.009 (3)	0.007 (3)	−0.002 (4)
C13	0.077 (4)	0.097 (6)	0.109 (7)	0.025 (5)	0.001 (4)	−0.004 (5)

### Geometric parameters (Å, º)

**Table:**

O1—C8	1.221 (7)	C4—H4A	0.9300
O2—C8	1.384 (8)	C5—C6	1.384 (10)
O2—C9	1.454 (6)	C5—H5A	0.9300
O3—C10	1.332 (6)	C6—H6A	0.9300
O3—C13	1.445 (7)	C7—C10	1.362 (7)
O4—C11	1.416 (7)	C7—C8	1.442 (8)
O4—H4	0.8200	C9—C10	1.464 (8)
O5—C12	1.424 (6)	C9—C11	1.539 (7)
O5—H5	0.8200	C9—H9	0.9800
C1—C6	1.379 (8)	C11—C12	1.507 (8)
C1—C2	1.392 (8)	C11—H11A	0.9800
C1—C7	1.462 (8)	C12—H12A	0.9700
C2—C3	1.367 (10)	C12—H12B	0.9700
C2—H2A	0.9300	C13—H13A	0.9600
C3—C4	1.355 (10)	C13—H13B	0.9600
C3—H3A	0.9300	C13—H13C	0.9600
C4—C5	1.362 (9)		
			
C8—O2—C9	108.0 (5)	O2—C9—C10	103.4 (5)
C10—O3—C13	120.1 (5)	O2—C9—C11	107.8 (5)
C11—O4—H4	109.5	C10—C9—C11	115.1 (5)
C12—O5—H5	109.5	O2—C9—H9	110.1
C6—C1—C2	116.3 (6)	C10—C9—H9	110.1
C6—C1—C7	122.3 (6)	C11—C9—H9	110.1
C2—C1—C7	121.5 (6)	O3—C10—C7	122.6 (6)
C3—C2—C1	122.3 (7)	O3—C10—C9	125.1 (5)
C3—C2—H2A	118.8	C7—C10—C9	112.3 (5)
C1—C2—H2A	118.8	O4—C11—C12	111.0 (5)
C4—C3—C2	120.4 (8)	O4—C11—C9	107.7 (5)
C4—C3—H3A	119.8	C12—C11—C9	111.5 (5)
C2—C3—H3A	119.8	O4—C11—H11A	108.8
C3—C4—C5	118.9 (8)	C12—C11—H11A	108.8
C3—C4—H4A	120.5	C9—C11—H11A	108.8
C5—C4—H4A	120.5	O5—C12—C11	108.6 (4)
C4—C5—C6	121.2 (7)	O5—C12—H12A	110.0
C4—C5—H5A	119.4	C11—C12—H12A	110.0
C6—C5—H5A	119.4	O5—C12—H12B	110.0
C1—C6—C5	120.8 (7)	C11—C12—H12B	110.0
C1—C6—H6A	119.6	H12A—C12—H12B	108.4
C5—C6—H6A	119.6	O3—C13—H13A	109.5
C10—C7—C8	105.2 (6)	O3—C13—H13B	109.5
C10—C7—C1	131.7 (6)	H13A—C13—H13B	109.5
C8—C7—C1	123.1 (6)	O3—C13—H13C	109.5
O1—C8—O2	117.1 (6)	H13A—C13—H13C	109.5
O1—C8—C7	132.0 (7)	H13B—C13—H13C	109.5
O2—C8—C7	110.8 (5)		
			
C6—C1—C2—C3	0.5 (10)	C8—O2—C9—C10	−5.6 (6)
C7—C1—C2—C3	−179.3 (6)	C8—O2—C9—C11	116.8 (5)
C1—C2—C3—C4	0.1 (12)	C13—O3—C10—C7	−168.3 (5)
C2—C3—C4—C5	−0.3 (12)	C13—O3—C10—C9	14.6 (8)
C3—C4—C5—C6	0.0 (12)	C8—C7—C10—O3	−179.8 (5)
C2—C1—C6—C5	−0.8 (9)	C1—C7—C10—O3	0.7 (9)
C7—C1—C6—C5	179.0 (6)	C8—C7—C10—C9	−2.4 (6)
C4—C5—C6—C1	0.6 (11)	C1—C7—C10—C9	178.2 (5)
C6—C1—C7—C10	−173.2 (6)	O2—C9—C10—O3	−177.6 (5)
C2—C1—C7—C10	6.6 (9)	C11—C9—C10—O3	65.0 (8)
C6—C1—C7—C8	7.5 (8)	O2—C9—C10—C7	5.0 (6)
C2—C1—C7—C8	−172.7 (6)	C11—C9—C10—C7	−112.4 (6)
C9—O2—C8—O1	−175.9 (5)	O2—C9—C11—O4	−66.2 (6)
C9—O2—C8—C7	4.6 (6)	C10—C9—C11—O4	48.7 (6)
C10—C7—C8—O1	179.2 (7)	O2—C9—C11—C12	55.9 (7)
C1—C7—C8—O1	−1.4 (10)	C10—C9—C11—C12	170.7 (5)
C10—C7—C8—O2	−1.4 (6)	O4—C11—C12—O5	−69.4 (6)
C1—C7—C8—O2	178.1 (5)	C9—C11—C12—O5	170.5 (6)

### Hydrogen-bond geometry (Å, º)

**Table:**

*D*—H···*A*	*D*—H	H···*A*	*D*···*A*	*D*—H···*A*
O4—H4···O5^i^	0.82	1.89	2.707 (5)	178
O5—H5···O1^ii^	0.82	1.94	2.741 (6)	165
C12—H12*B*···O4^iii^	0.97	2.58	3.365 (8)	139

Symmetry codes: (i) −*x*+1, *y*−1/2, −*z*+1; (ii) −*x*+2, *y*+1/2, −*z*+1; (iii) *x*, *y*+1, *z*.
