# Supplementary figures and images for: Crystal structure of (5R)-5-[(1S)-1,2-di­hydroxy­eth­yl]-4-meth­oxy-3-phenyl-2,5-di­hydro­furan-2-one
Source: Acta Crystallogr Sect E Struct Rep Online. 2014 Sep 30;70(Pt 10):o1141–2. doi: 10.1107/S1600536814021370 (PMC4257168; doi:10.1107/S1600536814021370)

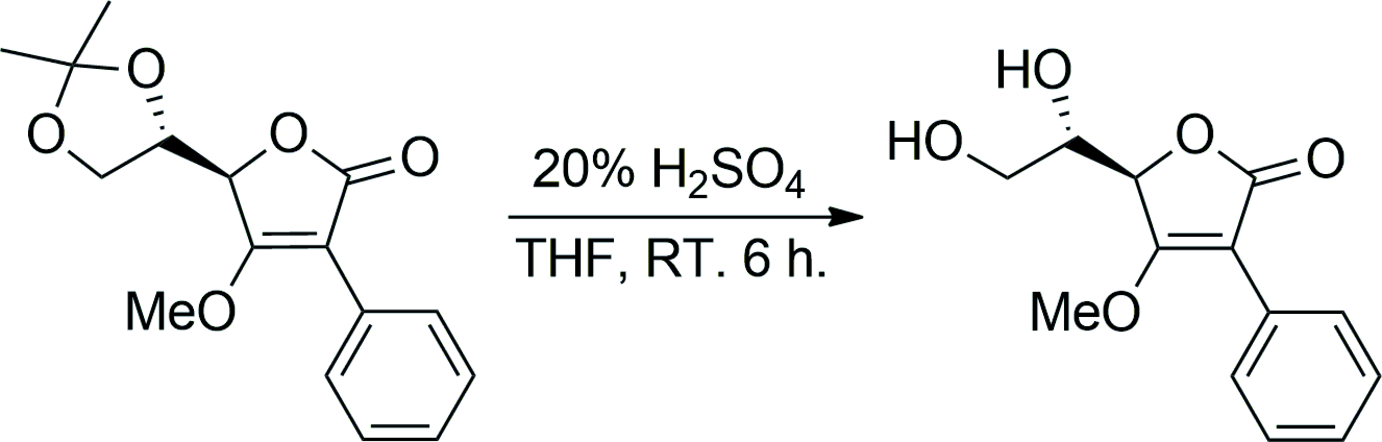

Supplement: Supplementary file 4 [file e-70-o1141-fig1.tif]

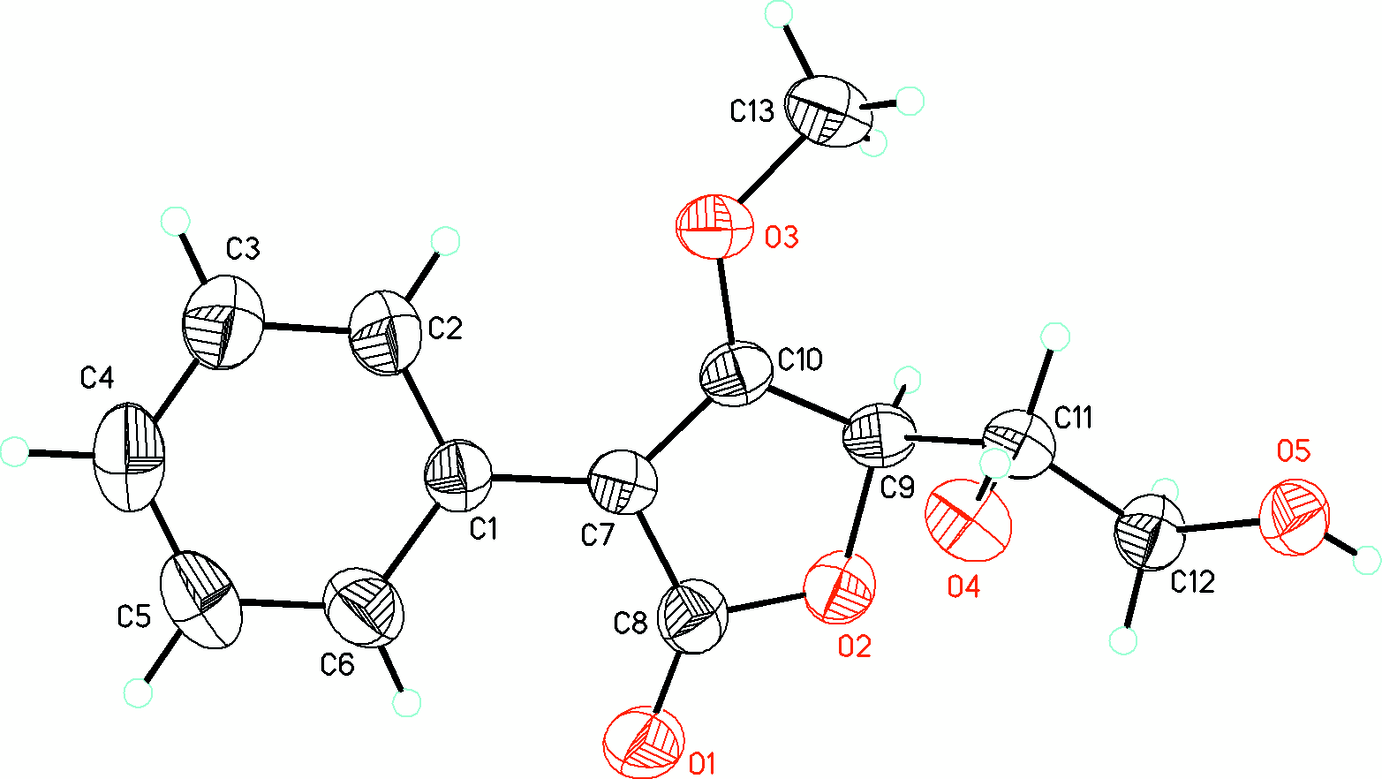

Supplement: Supplementary file 5 [file e-70-o1141-fig2.tif]

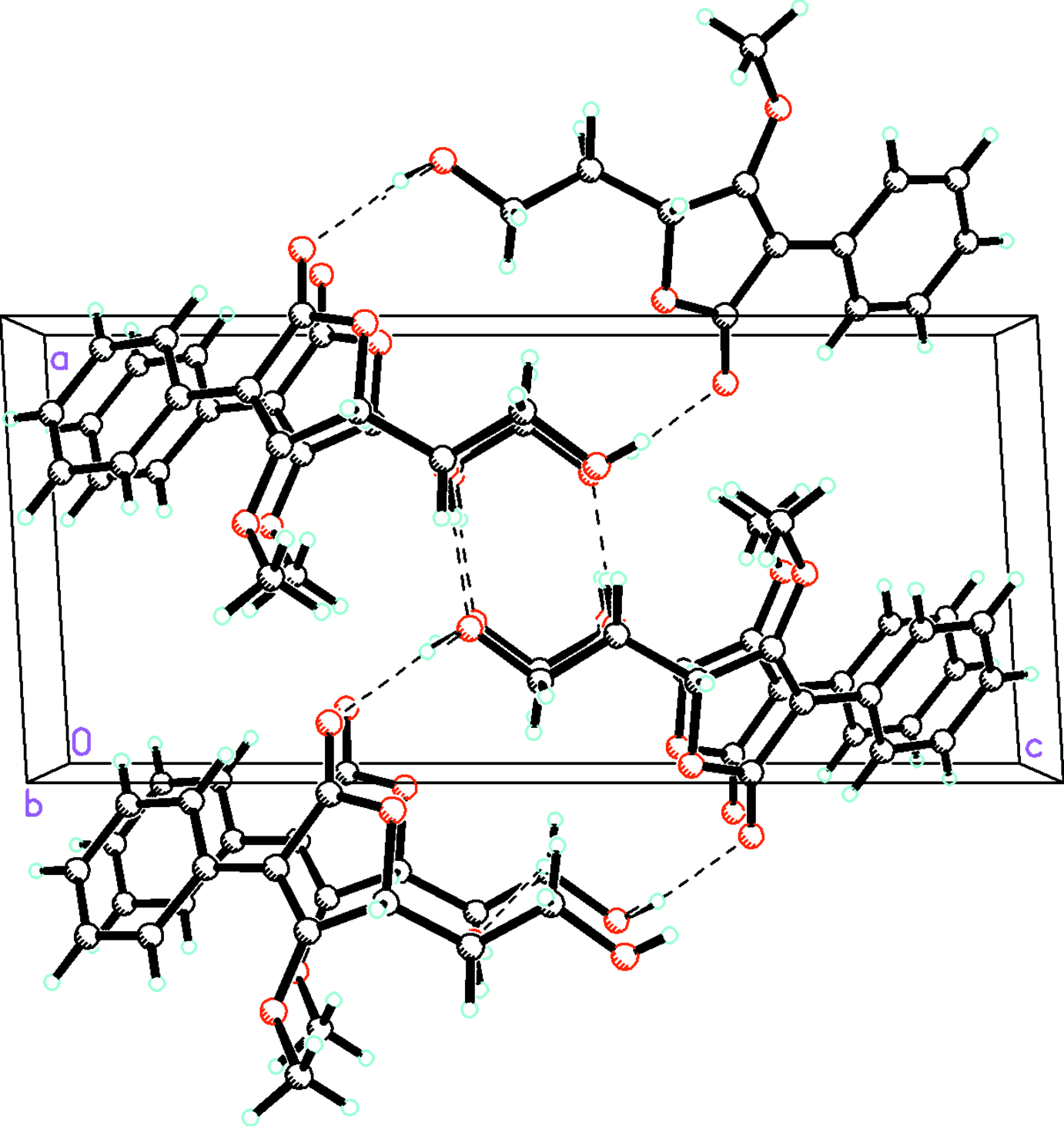

Supplement: Supplementary file 6 [file e-70-o1141-fig3.tif]
